# Delay in accessing definitive care for patients with microbial keratitis in Nepal

**DOI:** 10.3389/fmed.2022.915293

**Published:** 2022-07-22

**Authors:** Jeremy J. Hoffman, Reena Yadav, Sandip Das Sanyam, Pankaj Chaudhary, Abhishek Roshan, Sanjay K. Singh, Sailesh K. Mishra, Simon Arunga, Victor H. Hu, David Macleod, Astrid Leck, Matthew J. Burton

**Affiliations:** ^1^International Centre for Eye Health, London School of Hygiene and Tropical Medicine, London, United Kingdom; ^2^Sagarmatha Choudhary Eye Hospital, Lahan, Nepal; ^3^Nepal Netra Jyoti Sangh, Kathmandu, Nepal; ^4^Department of Ophthalmology, Mbarara University of Science and Technology, Mbarara, Uganda; ^5^MRC International Statistics and Epidemiology Group, London School of Hygiene and Tropical Medicine, London, United Kingdom; ^6^National Institute for Health Research Biomedical Research Centre for Ophthalmology at Moorfields Eye Hospital NHS Foundation Trust and UCL Institute of Ophthalmology, London, United Kingdom

**Keywords:** microbial keratitis, fungal keratitis, epidemiology, health systems, Nepal, cornea

## Abstract

**Background:**

The aim of this study was to describe the health-seeking journey for patients with microbial keratitis (MK) in Nepal and identify factors associated with delay.

**Methods:**

Prospective cohort study where MK patients attending a large, tertiary-referral eye hospital in south-eastern Nepal between June 2019 and November 2020 were recruited. We collected demographic details, clinical history, and examination findings. Care-seeking journey details were captured including places attended, number of journeys, time from symptom onset, and costs. We compared “direct” with “indirect” presenters, analyzing for predictors of delay.

**Results:**

We enrolled 643 patients with MK. The majority (96%) self-referred. “Direct” attenders accounted for only 23.6% (152/643) of patients, the majority of “indirect” patients initially presented to a pharmacy (255/491). Over half (328/643) of all cases presented after at least 7 days. The total cost of care increased with increasing numbers of facilities visited (*p* < 0.001). Those living furthest away were least likely to present directly (*p* < 0.001). Factors independently associated with delayed presentation included distance >50 km from the eye hospital [aOR 5.760 (95% CI 1.829–18.14, *p* = 0.003)], previous antifungal use [aOR 4.706 (95% CI 3.139–5.360)], and two or more previous journeys [aOR 1.442 (95% CI 1.111–3.255)].

**Conclusions:**

Most patients visited at least one facility prior to our institution, with time to presentation and costs increasing with the number of prior journeys. Distance to the eye hospital is a significant barrier to prompt, direct presentation. Based on these findings, improving access to eye care services, strengthening referral networks and encouraging early appropriate treatment are recommended to reduce delay, ultimately improving clinical outcomes.

## Introduction

Microbial keratitis (MK) is the leading cause of unilateral blindness after cataract in low- and middle-income countries (LMICs), previously estimated to be responsible for over 2 million cases of unilateral blindness annually in Asia and Africa (1). As blindness is usually defined as bilateral sight-loss, MK is often overlooked. However, MK leads to significant morbidity (2), reduced quality of life (3), and an associated high economic cost (4). There have been calls for MK to be recognized as a neglected tropical disease by the World Health Organization (5). The incidence of MK in Nepal is amongst the highest levels in the world, reported as 799/100,000/year (6).

MK can be caused by fungi (yeasts, molds and microsporidia), bacteria, viruses and protozoa (e.g., *Acanthamoeba spp*.), with filamentous fungi being more commonly implicated in tropical LMICs such as Nepal, where it accounts for up to 70% of cases, compared to bacterial keratitis which is more common in temperate latitudes (7, 8). It is an ophthalmic emergency, presenting with pain, photophobia, conjunctival hyperaemia, and corneal ulceration with a stromal inflammatory cell infiltrate. Effective treatment relies on promptly diagnosing and treating the patient with intensive antimicrobial agents. Any delay in presentation to appropriate eye care facilities allows the infection to become well-established, resulting in poor clinical outcomes (9, 10), with any treatment to improve this prognosis very challenging (11). Early application of topical antimicrobial agents following corneal abrasion prevents infection developing and allows for full recovery (12, 13).

A recent study from Nepal reported that patients referred to a tertiary level eye hospital in Kathmandu took on average 21.5 days to present from symptom onset, with 53% of cases directly presenting to a trained eye health worker or ophthalmologist at any clinical facility (including the tertiary hospital in Kathmandu) (14). However, this study did not investigate factors associated with delayed presentation. Our previous work from sub-Saharan Africa showed that delayed presentation is a key determinant of a poor outcome (9, 10, 15). There have been no published studies from Asia on associations with delay for MK patients.

Understanding the patient health-seeking journey can highlight gaps in the health system, helping direct resources into ensuring a rapid onward referral to appropriate care, with the goal of improving outcome for patients with MK before it is too late. The aim of this study was to describe the presentation journey of patients with MK to an eye hospital in south-eastern Nepal and to investigate factors associated with delayed presentation.

## Methods

### Ethical statement

This study followed the tenets of the Declaration of Helsinki. It was approved by the London School of Hygiene & Tropical Medicine Ethics Committee (Ref. 14841) and Nepal Health Research Council Ethical Review Board (Ref. 1937). Written informed consent in the local language was obtained before enrolment. If the patient was unable to read, the information was read to them, and they were asked to indicate their consent by application of their thumbprint, which was independently witnessed.

### Study design and setting

We prospectively recruited patients at Sagarmatha Choudhary Eye Hospital (SCEH) in Lahan, Nepal between 3rd June 2019 and 9th November 2020. It formed part of the triaging assessment used to enroll eligible patients with fungal keratitis (FK) into a randomized controlled trial comparing natamycin 5% to chlorhexidine 0.2%. The full protocol for this study has been published (16). SCEH is a tertiary ophthalmic referral hospital within Province 2 of south-eastern Nepal that serves a population of ~5 million people. It is located ~18 km from the Indian border, with many patients treated in outpatients being Indian nationals. There are 22 satellite “Eye Care Centers” (ECCs) located within Province 2 that are operated by SCEH and provide routine eye examination and treatment, referring to SCEH for more complex cases and surgery.

### Participants

Eligible patients were adults (>18 years) with acute MK, defined as having corneal epithelial ulceration >1 mm in diameter, corneal stromal infiltrate, and any/all signs of acute inflammation (conjunctival hyperaemia, anterior chamber inflammatory cells, hypopyon). All eligible patients who consented to participate in the study were included.

### Data collection procedures

Detailed demographic information was recorded. Clinical history data collected included date of symptom onset, detailed history of any preceding trauma and any prior treatment received, including traditional eye medicine (TEM) and/or conventional medications. A comprehensive “journey” history was obtained, using similar methodology to our previous work in Uganda (9). In brief, this comprised information on the number of journeys participants took prior to attending SCEH and their dates, the previous facilities visited, any previous medication used, and how much each step cost them in Nepali Rupees (transportation, consultation fees, medications). The complete patient “journey” ended when the patient presented to SCEH corneal clinic on the date of enrolment.

Global Positioning System (GPS) co-ordinates were generated for participants' residence using Google Maps and the closest patient-reported searchable landmark (e.g., village, school, health post). Straight-line distance from participants' home to SCEH were calculated from these co-ordinates using the haversine formula.

Clinical examination included best spectacle corrected visual acuity (BSCVA) in LogMAR and slit-lamp examination. The BSCVA protocol followed that used in the Steroids for Corneal Ulcers Trial (SCUT) (17), using a 3 m, proportionally-reduced version of the 4 m Early Treatment Diabetic Retinopathy Study tumbling “E” chart (Good-Lite, Illinois, USA) (18). Slit-lamp examination by a trial-certified ophthalmologist or ophthalmic assistant followed a structured approach: eyelid assessment, corneal ulcer features, anterior chamber characteristics (flare, cells, hypopyon shape, and size), and perforation status (16). Infiltrate and epithelial defect size was calculated as the mean of the maximum diameter of the infiltrate and the widest perpendicular diameter (19). *In vivo* confocal microscopy (IVCM) was performed prior to corneal sample collection. IVCM was performed by trained experienced operators using the HRT III/RCM confocal microscope (Heidelberg Engineering, Dossenheim, Germany) using a previously described technique (20, 21). All the images were reviewed during the procedure in real-time and classified into the various forms of keratitis, by one experienced observer. Corneal specimens were obtained for microbiological testing on site (16).

### Analysis

Data were analyzed in Stata 17 (Stata Corp., USA). Similar to our previous work, we classified participants into either “direct” or “indirect” presenters, depending on whether they received their definitive diagnosis and treatment at SCEH Corneal Clinic as their first point of care (9). Patients attending SCEH but who were not referred to the Corneal Clinic and therefore did not receive definitive care at their first visit, necessitating a second visit to SCEH (and their first visit to SCEH Corneal Clinic) were classed as indirect presenters. Summary frequency tables of demographics and clinical features were created with statistical testing performed using Wilcoxon rank sum for continuous variables and χ^2^ test for categorical variables. The geo-location of all known participants' home addresses were added to a custom map using the Google My Maps function (22). The patients' journeys from home to each facility, and the final journey from home to SCEH, was presented using median time intervals in days and interquartile ranges (IQRs). The cost of intermediate care was described by summarizing the total patient expenditure for each journey (consultation cost, travel cost and any medication cost, where applicable) and presented as median expenditure with IQRs in Nepal Rupees. The Cuzick non-parametric test for trend was used to test for an association between expenditure and the number of facilities visited.

Time from symptom onset until presentation at SCEH (presentation time) was divided into the following categories for analysis of “delay”: “prompt” (0–3 days), “early” (4–7 days), “intermediate” (8–14 days), “late” (15–30 days), and “very late” (>30 days). Any visit other than “prompt” (i.e., four or more days after symptom onset) can be considered as delayed presentation (10). Ordered logistic regression was performed to determine the factors associated with these five categories of “delay,” whilst pairwise associations between clinical or demographic factors and direct presentation were investigated using univariable logistic regression to estimate crude odds ratios (OR). We took a causal modeling approach to explore the association of different potential risk factors for delayed presentation or indirect attendance. The risk factors investigated are given in Supplementary Material 1. To help inform our modeling, we mapped out relationships between different variables to identify those to adjust for to determine the overall effect of the exposure on the outcome. A change in point estimate criteria was used to assess for confounding; if the log odds ratio changed by more than 10% we adjusted for that in the model. The model was further checked to identify any collinearity by reviewing uncentered variance inflation factors (VIF). If the VIF was >10, then it was deemed to suggest collinearity and therefore the confounding variable removed from the model. This process was repeated until all VIF values were acceptable. Adjusted OR were reported for the final model.

## Results

### Demographic features

We triaged 890 consecutive patients with suspected microbial keratitis, of which we enrolled 643 patients. We excluded 247 cases as follows: 144 did not consent or were children, 95 had a healed or chronic corneal ulcer, 5 attended out-of-hours, and 3 cases were not microbial keratitis. Only 152/643 (23.6%) of patients were direct presenters. The remaining 491/643 (76.4%) were indirect presenters, including 6 patients who initially visited SCEH but required a second journey and return visit to SCEH to receive a definitive diagnosis and treatment. Demographic and clinical features of direct vs. indirect presenters are shown in Table 1. Direct presenters lived closer to SCEH (median distance 24.5 vs. 40.2 km, *p* < 0.001) and lived closer to a health center (median distance 1 vs. 2 km, *p* < 0.001). A higher proportion of direct presenters were Nepali (74.3%), compared to indirect presenters (53.2%, *p* < 0.001). A greater proportion of direct presenters were farmers (61.2%) compared to indirect presenters (48.7%, *p* = 0.026). The two groups were otherwise similar for the other variables investigated, including age, gender, education, marital status, and literacy. Most patients from India lived further away than patients from Nepal; 216/272 (79.4%) of Indian patients lived more than 50 km from SCEH, compared to 42/271 (15.5%) of Nepali patients.

**Table 1:** Baseline characteristics of direct vs. indirect presenters, *n* = 643.

		**Total (*****n*** = **643)**			**Direct presenters (*****N*** = **152)**			**Indirect presenters (*****N*** = **491)**			
**Continuous variables**		**Median**	**(IQR)**	**(Range)**	**Median**	**(IQR)**	**(Range)**	**Median**	**(IQR)**	**(Range)**	***P*** **-value**
Age		45.9	(35.7–57.7)	(18.1–100.1)	50.1	(35.8–60.6)	(18.9–75.8)	45.9	(35.6–55.9)	(18.1–100.1)	0.1450
Distance to SCEH, km^a^		37.3	(23.5–66.2)	(0.09–756.1)	24.5	(14.9–51.0)	(0.9–282.1)	40.2	(27.1–74.6)	(0.09–756.1)	<0.001
Distance to nearest health center, km^**a**^		2	(1–5)	(0–50)	1	(0–3)	(0–40)	2	(1–5)	(0–50)	<0.001
**Categorical variables**		**N**	(%)		**(N)**	(%)		**N**	(%)		***P* value**
Gender	Female	392	(61.0)		94	(61.8)		298	(60.7)		
	Male	251	(39.0)		58	(38.2)		193	(39.3)		0.849
Occupation	Agriculture	332	(51.6)		93	(61.2)		239	(48.7)		
	No job	263	(40.9)		49	(32.2)		214	(43.6)		
	Non-agriculture	48	(7.5)		10	(6.6)		38	(7.7)		0.026
Marital status	Married	577	(89.7)		140	(92.1)		437	(89.0)		
	Unmarried^b^	66	(10.3)		12	(7.9)		54	(11.0)		0.358
Literacy (read/write)	Illiterate	500	(77.8)		115	(75.7)		385	(78.4)		
	Little Nepali	51	(7.9)		16	(10.5)		35	(7.1)		
	Nepali well	48	(7.5)		12	(7.9)		36	(7.3)		
	English and Nepali	44	(6.8)		9	(5.9)		35	(7.1)		0.542
Country of residence	Nepal	371	(57.7)		113	(74.3)		258	(52.6)		
	India	272	(42.3)		39	(25.7)		233	(47.5)		<0.001
Education	None	494	(76.8)		114	(75.0)		380	(77.4)		
	Primary level	80	(12.4)		21	(13.8)		59	(12.0)		
	Secondary level	12	(1.9)		3	(2.0)		9	(1.8)		
	Tertiary level	57	(8.9)		14	(9.2)		43	(8.8)		0.897
Where first presented	Pharmacy	255	(39.7)		0	(0)		255	(51.9)		
	Health post	6	(0.9)		0	(0)		6	(1.2)		
	Private clinic	93	(14.5)		0	(0)		93	(18.9)		
	Government hospital	12	(1.9)		0	(0)		12	(2.4)		
	Private hospital	24	(3.7)		0	(0)		24	(4.9)		
	Traditional healer	1	(0.16)		0	(0)		1	(0.2)		
	Eye care clinic (ECC)	94	(14.6)		0	(0)		94	(14.6)		
	SCEH	158	(24.6)		152	(100)		6	(1.22)		<0.001

SCEH, Sagarmatha choudhary eye hospital.

a Variables with some missing data: distance to SCEH [n = 635, (direct 154)], distance to nearest health center [n = 641, (direct 155)].

b Unmarried included single, divorced, and widowed.

There were some interesting differences between direct and indirect presenters in terms of clinical features, Table 2. Indirect presenters had a longer median presentation time from symptom onset (8 vs. 5 days, *p* < 0.001), worse vision (0.6 vs. 0.3 logMAR, *p* < 0.001) and larger corneal ulcers [median infiltrate and epithelial defect size 2.9 vs. 2.1 mm (*p* < 0.001) and 3.0 vs. 2.5 mm (*p* < 0.001), respectively], compared to direct presenters. The proportion of patients who had used treatment prior to presenting at SCEH was significantly higher for indirect presenters (98.4%) compared to direct presenters (44.1%, *p* < 0.001). This held true for all forms of conventional medication (*p* < 0.001). The numbers of patients who had used traditional eye medicines was low overall (12/643, 1.9%), with proportionally more in the indirect vs. direct group but not statistically significant (*p* = 0.739). There were proportionally more direct presenters with a diagnosis of bacterial keratitis (9.9%) compared to indirect presenters (3.5%), and conversely more indirect presenters with a diagnosis of fungal keratitis (77.4%) compared to direct presenters (67.6%, *p* = 0.003). There was no evidence of a difference in rates of trauma between direct and indirect presenters, although farmers were more likely to have a history of trauma compared to non-farmers (*p* < 0.001, Supplementary Table 1).

**Table 2:** Clinical history and clinical signs of direct vs. indirect presenters (*n* = 643).

		**Total (*****n*** = **643)**			**Direct presenters (*****N*** = **152)**			**Indirect presenters (*****N*** = **491)**			
**Continuous variables**		**Median**	**(IQR)**	**(Total range)**	**Median**	**(IQR)**	**(Total range)**	**Median**	**(IQR)**	**(Total range)**	***P*** **-value**
Presentation time, days^a^		8	(5–14)	(1–92)	5	(3–10)	(1–92)	8	(5–15)	(1–82)	<0.001
Presenting vision (LogMAR)		0.54	(0.2–1.4)	(0–1.9)	0.3	(0.08–0.89)	(0–1.9)	0.6	(0.24–1.5)	(0–1.9)	<0.001
Infiltrate size, mm^b^		2.75	(1.75–4)	(0.2–11.75)	2.1	(1.5–3.5)	(0.5–8.8)	2.9	(1.9–4.1)	(0.2–11.8)	<0.001
Epithelial defect size, mm^b^		2.9	(2–4.25)	(0–12)	2.5	(1.9–3.8)	(0.6–9)	3.0	(2.1–4.4)	(0–12)	<0.001
**Categorical variables**		**N**	(%)		**(N)**	(%)		**N**	(%)		***P* value**
Presenting time	Prompt (0–3 days)	86	(13.4)		42	(27.6)		44	(9.0)		
	Early (4–7 days)	229	(35.6)		54	(35.5)		175	(35.6)		
	Intermediate (8–14 days)	180	(28.0)		33	(21.7)		147	(29.9)		
	Late (15–30 days)	108	(16.8)		19	(12.5)		89	(18.1)		
	Very late (>30 days)	40	(6.2)		4	(2.6)		36	(7.3)		<0.001
History of trauma	None/unsure	326	(50.7)		72	(47.4)		254	(51.7)		
	Vegetative matter	226	(35.2)		64	(42.1)		162	(33.0)		
	Other	86	(13.4)		15	(9.9)		71	(14.5)		
	Unknown object	5	(0.8)		1	(0.7)		4	(0.8)		0.153
Previous treatment^c^	No	93	(14.5)		85	(55.9)		8	(1.6)		
	Yes	550	(85.5)		67	(44.1)		483	(98.4)		<0.001
	Steroids	105	(16.3)		12	(7.9)		93	(18.9)		<0.001
	Antibiotics	463	(72.0)		54	(35.5)		409	(83.3)		<0.001
	Antifungals	134	(20.8)		8	(5.8)		125	(25.7)		<0.001
	Other topical	260	(40.4)		18	(11.8)		242	(49.3)		<0.001
	Systemic medication	353	(54.9)		29	(19.1)		324	(66.0)		<0.001
	TEM	12	(1.9)		2	(1.3)		10	(2.0)		0.741
Most important symptom	Pain	471	(73.3)		115	(75.7)		356	(72.5)		
	Vision	57	(8.9)		13	(8.6)		44	(9.0)		
	Other	115	(17.9)		24	(15.8)		91	(18.5)		0.739
Hypopyon^d^	No	457	(71.2)		112	(74.2)		345	(70.3)		
	Yes	175	(27.3)		35	(23.2)		140	(28.5)		
	Unable to see	10	(1.6)		4	(2.7)		6	(1.2)		0.223
Perforation status^d^	No	634	(98.8)		151	(100)		483	(98.4)		
	Descemetocele	6	(0.9)		0	(0)		6	(1.2)		
	Perforated	2	(0.3)		0	(0)		2	(0.4)		0.483
Diagnosis^d^	Fungal keratitis	482	(75.1)		102	(67.6)		380	(77.4)		
	Bacterial keratitis	32	(5.0)		15	(9.9)		17	(3.5)		
	Mixed	51	(7.9)		18	(11.9)		33	(6.7)		
	Unknown	77	(12.0)		16	(10.6)		61	(12.4)		0.003

*TEM, traditional eye medicine*.

a Presentation time was measured as duration in days it took to come to the eye hospital after onset of symptoms.

b geometrical of the largest diameter and the diameter perpendicular to the largest diameter.

c Previous treatment was often dispensed by local pharmacies without a prescription or clinician review; by definition this was the case for all “direct” attenders.

d One patient was not able to be examined.

### Factors associated with direct presentation

Distance from SCEH and residence in India were associated with reduced odds of direct presentation in the univariable analysis (Table 3), whilst being a farmer was associated with increased odds of direct presentation. Both these variables remained as significant independent associations in the multivariable model. Country of residence was removed as this was collinear with distance from SCEH.

**Table 3:** Univariable and multivariable logistic regression analysis of factors associated with direct presentation to the eye hospital, *n* = 643.

**Variable**	**Univariable analysis**			**Multivariable analysis**		
	**cOR^c^**	**(95% CI)**	***P*** **-value**	**aOR^c^**	**(95% CI)**	***P*** **-value**
Age	1.008	(0.995–1.022)	0.218			
Sex (being female)	1.050	(0.722–1.526)	0.800			
Marital status (being married)	1.442	(0.750–2.772)	0.273			
Farmer occupation	1.662	(1.147–2.408)	0.007	1.535	(1.024–2.30)	0.038
Distance to SCEH (km)						
0–5	–	–	–			
5–20	0.641	(0.240–1.715)	0.376	0.587	(0.215–1.601)	0.298
20–50	0.173	(0.066–0.453)	<0.001	0.167	(0.063–0.447)	<0.001
50–100	0.145	(0.053–0.397)	<0.001	0.137	(0.049–0.382)	<0.001
>100	0.116	(0.040–0.338)	<0.001	0.116	(0.039–0.349)	<0.001
Distance from nearest health center (km)						
0–12.5	–	–	–			
>12.5–25	0.750	(0.248–2.263)	0.609			
>25–37.5	0.354	(0.044–2.818)	0.327			
>37.5–50	1.593	(0.143–17.70)	0.705			
Positive history of trauma^a^	1.047	(0.710–1.543)	0.816			
Used TEM	0.641	(0.139–2.959)	0.569			
Education status						
None	–	–	–			
Primary level	1.186	(0.691–2.036)	0.535			
Secondary level	1.111	(0.295–4.173)	0.876			
Tertiary level	1.085	(0.575–2.055)	0.802			
Country of residence (India)^b^	0.382	(0.255–0.572)	<0.001			

*SCEH, Sagarmatha choudhary eye hospital; TEM, traditional eye medicine; cOR, crude odds ratio; aOR, adjusted odds ratio*.

a Definite history of trauma only; patients who were unsure were included in the no history of trauma group for the purpose of this analysis.

b Country of residence was removed from the multivariable regression model due to being collinear.

c OR <1 means they were less likely to present directly to the eye hospital.

Sensitivity analyses performed for patients living in India and Nepal separately found that for Nepali residents, distance >20 km was independently associated with reduced odds of direct presentation (Supplementary Table 2). However, there was no evidence of a similar association for Indian residents.

### Care-seeking pathway

**Figure 1:**
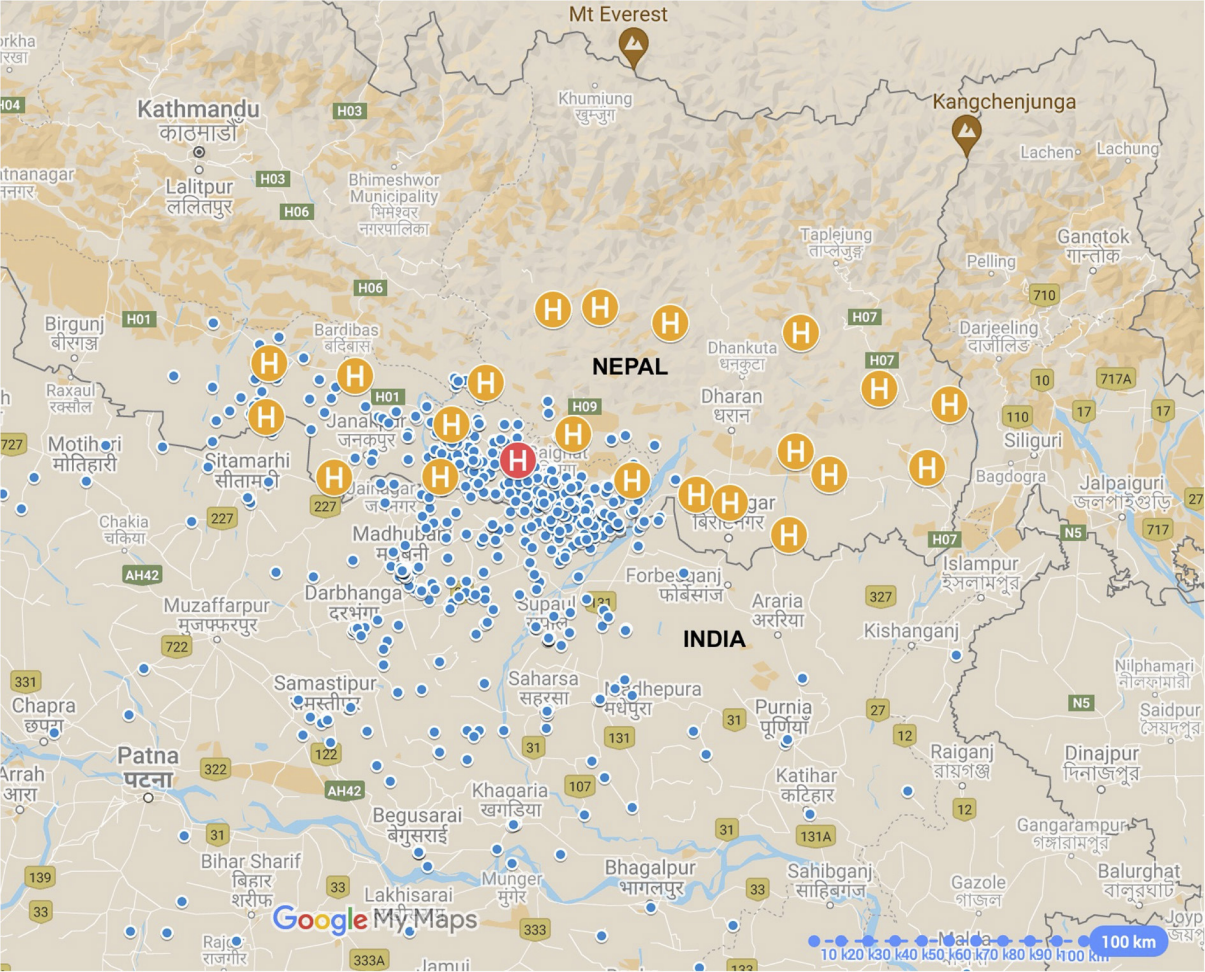
Map of eastern Nepal and north-eastern India showing patients' homes (blue pins) in relation to Sagarmatha Choudhary Eye Hospital (SCEH, red “H” pin) and Eye Care Centers (Orange “H” pin”). Not all positions are shown as some patients attended from outside of this map area. International borders shown as solid gray lines.

Figure 1 shows the locations of the participants' homes, SCEH and the satellite eye care centers. Most patients (75%) lived within 66 km of the eye hospital, clustered within Province 2 of Nepal and from neighboring Bihar state in Northern India (both low-land, sub-tropical plains areas). However, 15% of patients traveled more than 100 km to attend SCEH, mostly from the Indian states of Assam and West Bengal in north-eastern India.

Table 1 identifies the type of facility where patients first presented. Pharmacies were the most frequent, followed by direct presentation to SCEH, satellite ECC clinics and private clinics. Only one patient reported visiting a traditional healer first. Pharmacies were visited much less frequently on subsequent visits: only 6/491 (1.2%) of second journey destinations were pharmacies, and 1/103 (0.97%) of third journey destinations. There were no visits to pharmacies beyond this. Only 39/272 (14.3%) of Indian patients attended SCEH directly.

**Figure 2:**
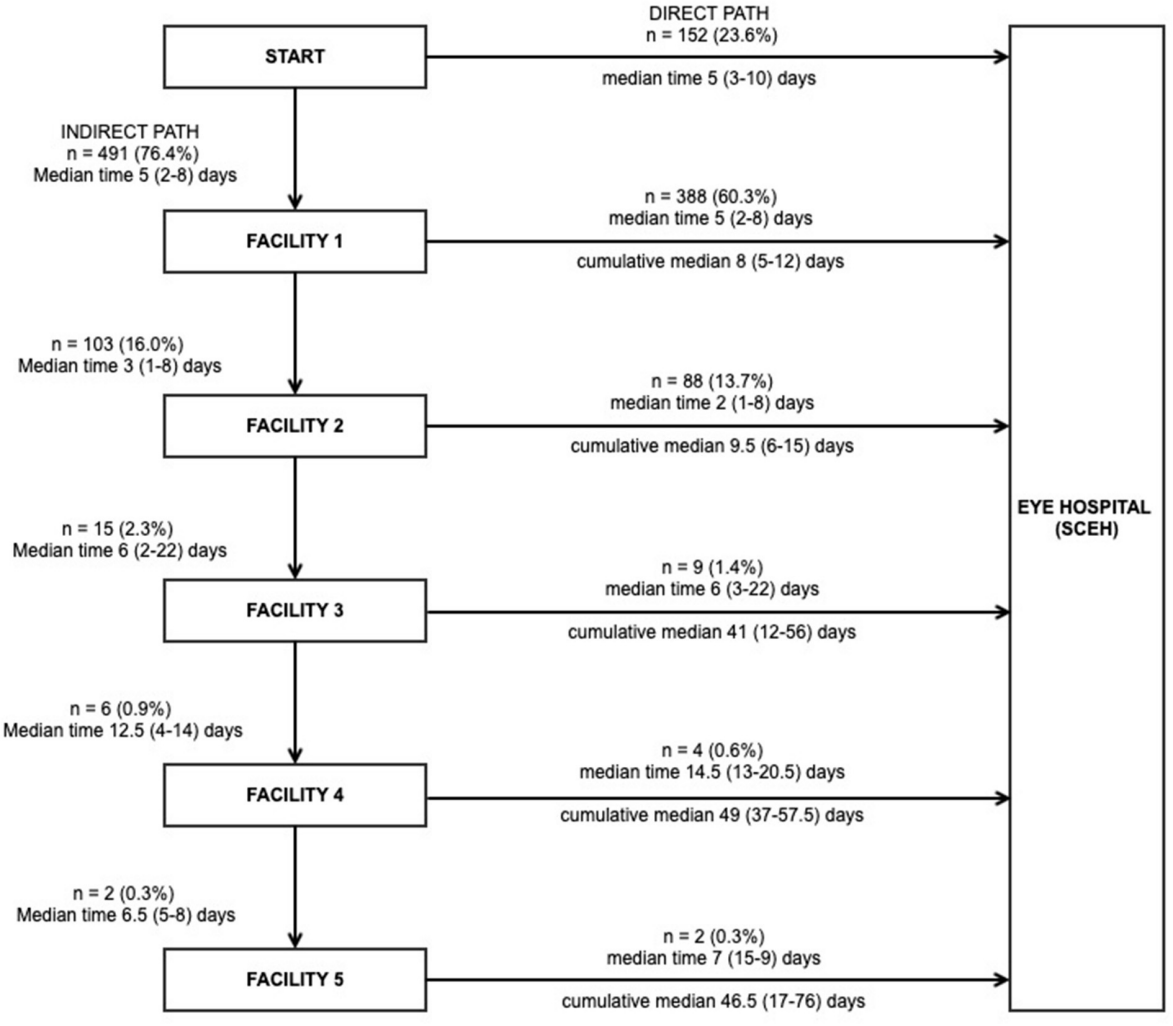
The care-seeking journey of patients with microbial keratitis, the time taken at each stage, and the cumulative time from onset of symptoms to presentation (*n* = 643). Note all patients returned home after attending each facility, from where they restarted any subsequent journeys, apart from the following exceptions: (1) Journey 2: one patient referred directly from a private clinic to a private hospital; seven patients referred directly from a satellite eye care clinic (ECC) to Sagarmatha Choudhary Eye Hospital (SCEH); (2) Journey 3: 19 patients referred directly from ECC to SCEH. Six patients attended SCEH as their first facility but did not receive a definitive diagnosis and/or treatment at that point and all had to make one additional journey to SCEH for final definitive treatment.

Figure 2 outlines the stages in the journey of patients from home to each intermediate facility, as well as their final journey for diagnosis and treatment at SCEH, including median times for each stage and cumulative median time from symptom onset. Nearly all patients (95.8%) returned home after visiting each facility and then made a subsequent journey; there were only 27 onward referrals between facilities (1 from a private hospital to a private clinic as an interim journey, and 26 from ECCs to SCEH as a final journey). The majority of patients (388/643, 60.3%) attended one facility (i.e., two journeys) before definitive treatment at SCEH, whilst 88/643 (13.7%), 9/643 (1.4%), 4/643 (0.6%), and 2/643 (0.3%) made three, four, five or six journeys, respectively. On average patients spent almost 1 week between visiting each facility.

Of the patients who had used steroids prior to attendance at SCEH, 64/105 (61%) had attended a pharmacy at one stage during their journey to SCEH, compared to 193/538 (35.9%) of patients who had no history of steroid use (*p* < 0.001).

### Cost of care

Table 4 presents the cost of care in Nepali Rupees (NPR). The total cost of care increased with additional facility visits. This is supported by evidence to suggest an association between the total cost and number of visits made (Cuzick non-parametric test for trend *p* < 0.0001). Direct presenters spent the least overall [median NPR 760 (IQR 620–900)]. Most of the expenditure was on consultations, followed by transportation, with medicine costs accounting for the smallest component of expenditure.

**Table 4:** Money spent by patients per number of facilities visited before coming to the eye hospital.

**Median cost of care (IQR) in Nepali Rupees** ^a^										
**Number of journeys**	***n***	**(%)**	**Transportation**		**Consultation**		**Medicine**		**Total expenditure**	
1^b^	152	(23.6)	240	(120–400)	480	(480–480)	0	(0–20)	760	(620–900)
2	388	(60.3)	320	(200–600)	530	(480–960)	38	(20–220)	1,055	(764–1,502)
3	88	(13.7)	415	(275–710)	560	(560–980)	220	(20–301)	1,325	(980–2,120)
4	9	(1.4)	850	(700–1,000)	1,480	(1,060–2,480)	24	(20–284)	2,718	(1,740–3,440)
5	4	(0.6)	1,965	(895–2,875)	2,480	(2,480–2,730)	515.5	(453.5–593)	5,038	(3,881–6,146)
6	2	(0.3)	830	(450–1,210)	1,205	(880–1,530)	464	(428–500)	2,499	(1,758–3,240)
*P*-value for test of trend										<0.001

a All costs are quoted in Nepali Rupees. The US $ exchange rate on 10/09/2021 was US$ 1 = 118.12.

b Patients with 1 journey were direct presenters to the hospital whose only consultation cost was the fixed cost for opening a file at the facility. As patients were seen as part of this study, most medicine costs were covered by the hospital.

### Factors associated with delay

The association between delay in presentation and multiple risk factors was modeled using univariable and multivariable ordered logistic regression (Table 5). We found evidence of independent associations between the following variables and presentation delay: distance from home to SCEH >50 km, visiting more than one facility prior to SCEH, prior treatment, previous use of antifungals, systemic medication and/or other topical medications, prior use of TEM, increasing number of journeys and residence in India. Distance from the nearest health center to home, prior use of topical antibiotics, and prior use of topical steroids, although significant on univariable analysis, were not associated with delay after adjustment. Conversely, there was evidence that higher educational achievement was associated with reduced odds of delay.

**Table 5:** Univariable and multivariable ordinal logistic regression analysis of factors associated with delay among patients with microbial keratitis (*n* = 643).

**Variable**	**Univariable analysis**			**Multivariable analysis**		
	**cOR**	**(95% CI)**	***P*** **-value**	**aOR**	**(95% CI)**	***P*** **-value**
Age	1.002	(0.992–1.013)	0.631			
Sex (being female)	0.981	(0.730–1.320)	0.903			
Marital status (being married)	0.844	(0.536–1.333)	0.468			
Farmer occupation	0.870	(0.651–1.162)	0.346			
Distance to SCEH (km)						
0–5	–	–	–	–	–	–
>5–20	0.896	(0.300–2.675)	0.843	1.132	(0.343–3.733)	0.839
>20–50	2.172	(0.776–6.078)	0.140	2.152	(0.694–6.675)	0.184
>50–100	8.069	(2.835–22.97)	<0.001	5.760	(1.829–18.14)	0.003
>100	13.37	(4.567–39.17)	<0.001	9.665	(2.974–31.41)	<0.001
Distance from nearest health center (km)						
0–12.5	–	–	–			
>12.5–25	2.024	(0.934–4.387)	0.074	1.259	(0.555–2.856)	0.581
>25–37.5	5.675	(1.745–18.46)	0.004	1.991	(0.575–6.891)	0.277
>37.5–50	4.680	(0.626–35.00)	0.133	1.165	(0.140–9.681)	0.887
Positive history of trauma^a^	0.872	(0.637–1.194)	0.394			
Education status						
None	–	–	–	–	–	–
Primary level	0.942	(0.606–1.464)	0.790	0.743	(0.441–1.254)	0.266
Secondary level	0.478	(0.143–1.596)	0.230	0.180	(0.045–0.720)	0.015
Tertiary level	0.434	(0.247–0.763)	0.004	0.272	(0.135–0.549)	<0.001
Country of residence (India)	5.205	(3.800–7.131)	<0.001	3.406	(2.417–4.800)	<0.001
Previous treatment	2.936	(1.835–4.696)	<0.001	2.068	(1.101–3.882)	0.024
Previous steroids	1.987	(1.357–2.909)	0.0004	1.580	(0.910–2.752)	0.106
Previous antibiotics	1.452	(1.045–2.019)	0.026	1.017	(0.683–1.516)	0.933
Previous antifungals	6.799	(4.685–9.865)	<0.001	4.706	(3.139–5.360)	<0.001
Previous other topical medication	1.778	(1.322–2.391)	0.0001	1.455	(1.027–2.061)	0.035
Previous systemic medication	3.610	(2.650–4.919)	<0.001	1.972	(1.352–2.878)	<0.001
Used TEM	2.603	(0.951–7.128)	0.063	2.512	(1.746–3.615)	<0.001
Number of journeys						
1	–	–	–	–	–	–
2	1.797	(1.239–2.608)	0.002	1.442	(0.968–2.1491)	0.072
3	2.976	(1.798–4.928)	<0.001	1.902	(1.111–3.255)	0.019
4	27.83	(6.774–114.4)	<0.001	11.80	(2.844–48.93)	0.001
Visiting one or more facilities prior to SCEH	2.099	(1.462–3.015)	<0.001	1.617	(1.096–2.386)	0.016

Ordered categories of delay to presentation from symptom onset: prompt (0–3 days), early (4–7 days), intermediate (8–14 days), late (15–30 days) and very late (>30 days).

a Definite history of trauma only; patients who were unsure were included in the no history of trauma group for the purpose of this analysis. SCEH, Sagarmatha choudhary eye hospital; TEM, traditional eye medicine; cOR, crude odds ratio; aOR, adjusted odds ratio.

Sensitivity analyses performed for patients living in India and Nepal separately found that that for Nepali residents, distance >50 km was independently associated with increased odds of delayed presentation (Supplementary Table 3). However, there was no evidence of a similar association for Indian residents. However, Indian residency remained a risk factor for delayed presentation in a multivariable model that only included patients living more than 20 km away from the hospital (OR 2.535 95% CI 1.343–4.788 *p* = 0.004).

## Discussion

This study describes the care-seeking journey of people with microbial keratitis in lowland Nepal and investigates factors associated with delayed presentation. This has highlighted several key issues, which are opportunities for intervention to improve care and outcomes.

We found that in our cohort of 643 patients, <1 quarter of patients (23.6%) attended the tertiary-level eye hospital directly, with the majority (60.3%) attending one facility beforehand. There were very few direct referrals from primary care providers; ECCs were responsible for the only onward referral of MK patients to SCEH in our study, with all other patients self-referring. Most patients (51%) presented to SCEH more than a week after symptom onset. As expected, patients living 20 km away or further were less likely to present directly (*p* < 0.001), whilst interestingly farmers were more likely to present directly (*p* = 0.036). Trauma was found to be more common in farmers than non-farmers, so a traumatic mechanism may have prompted farmers to attend sooner, although trauma itself was not associated with indirect presentation. This finding is contrary to work from Ghana that found farmers more likely to sustain trauma but less likely to make use of eye care facilities (23).

We found several variables to be independently associated with delayed presentation, with the greatest odds ratios for delay being distance from home to the eye hospital of 50 km or more, previous use of antifungals and four previous journeys. The further a person was from the point-of-care the greater the delay, as not only does travel become logistically more challenging, but also more expensive, meaning patients may not travel until they have exhausted easier options available locally. Furthermore, awareness of services offered by SCEH may be reduced the further someone is from the hospital. It is likely that the prior use of antifungals is associated with delayed presentation as it will have taken patients time and additional visits to other facilities to finally obtain this treatment. Conversely, we found higher than primary level education (*p* = 0.015) to be associated with prompt attendance, possibly due to improved health-awareness and health-seeking behavior as previously described in Nepal (24).

As expected, we found most patients to be clustered around SCEH on both sides of the Indian-Nepali border, with 75% living within 66 km of the eye hospital. However, 15% of patients traveled more than 100 km to attend SCEH, with one patient traveling more than 750 km. Nearly all patients were from low-land plain areas, with only a handful coming from hilly or mountainous locations, which are sparsely populated with limited agricultural activity. Living 50 km or further from SCEH was associated both with delayed and indirect presentations, remaining true with Indian-resident patients excluded. Conversely, Indian residence remained a risk factor for delayed presentation when only including patients living more than 20 km away from SCEH. These findings suggest that distance, as well as the international border, are significant barriers to prompt, direct attendance. A significant proportion of patients were from India (42.3%), greater than previously reported between 2010 and 2014 (16%) (25). The nearest eye hospitals within Bihar are in the state capital Patna, 180 km from SCEH. The lack of ophthalmic care within the northern part of Bihar state is likely the main driving force for Indian patients to attend SCEH, with the vast majority (84.5%) attending one or more facilities prior to SCEH.

The median time from symptom onset to attending the first facility was 5 days for both indirect and direct presenters, with the interval between subsequent journeys about 1 week on average. This is similar to the mean symptom-presentation interval to the first facility of 5.6 days reported elsewhere in Nepal (14), but a little slower than the 2-day interval reported in Uganda (9).

Previous work from Uganda found that TEM use and visiting one or more facilities prior to attendance were independently associated with delay to the eye hospital for patients with MK (9), similar to findings for other serious eye conditions in the African region (26–28). In contrast to this, we found TEM was used very infrequently (1.87%). Similarly, only one patient initially visited a traditional healer, in stark contrast to our experience in Uganda (9).

The total cost of care increased with increasing numbers of facilities visited (*p* < 0.001), consistent with previous studies (9, 29). Cost can be a major barrier to accessing eye health services (30). Consultation fees accounted for the largest component of overall expenditure, followed by transport and medications. This contrasts with Uganda, where the order was reversed due to government subsidies for health services (9). Medications are relatively cheap in Nepal, as most are generics manufactured in India and therefore easily imported at relatively affordable cost. Although there is currently limited literature concerning the economic burden of microbial keratitis in LMICs, it is likely that there are significant direct (cost of care, medicines, transport etc.) and indirect costs (lost earnings and assistance from carers). Studies from the USA and UK found increasing direct and indirect costs with increasing disease duration (31, 32), whilst in India patients who lived further away had a delayed presentation and spent more than those nearby (29). The additional costs incurred due to convoluted health-seeking journeys and the related delayed presentation add additional expenses to patients who are already under a significant financial pressure due to MK. By improving access to ophthalmic services and reducing the delay, these additional costs can be reduced.

This study highlights several areas for intervention to reduce delay to accessing eye care. A first opportunity is the initiation of appropriate early treatment and avoiding harmful treatment in community and primary care settings. Pharmacists were the first point of contact for many patients (39.7%). Pharmacists in Nepal are loosely regulated and can dispense most eye drops and oral antibiotics without a prescription. We found that they frequently dispensed steroid eye drops. Steroids can mask clinical signs and suppress the immune response, resulting in worse outcomes, particular for fungal infections (7). If pharmacists can be trained to avoid using steroids, dispense topical antibiotics alone, and refer urgently to an eye hospital, then delay might be reduced and outcomes improved. Pharmacists should be seen as an integral part of primary ophthalmic care and given the training and resources required to support this.

A second opportunity for intervention is improving access to primary eye care facilities. Given distance from the eye hospital is a significant risk factor for delayed presentation, such delays could potentially be mitigated by improved access to primary eye care services or through satellite hospital clinics (as in the case of ECCs for SCEH). There are currently no ECCs in the region of India where most of the Indian patients come from. Introducing such facilities, which could refer to SCEH or equivalent institutions in India, would be expected to reduce the presentation time for Indian patients. Increasing awareness through advertising or media campaigns amongst the northern Bihar population to attend SCEH in the event of symptoms suggestive of MK may further improve access. Co-operation on a regional scale between local governments in both Nepal and India may help reduce logistical barriers by improving transport links and streamlining border crossings.

The knowledge and skills amongst primary healthcare workers (PHCWs) in Eastern Nepal where our study was conducted have been shown to be inadequate to provide quality primary eye care services (33). For example, only 8.4% of 107 PHCWs surveyed across 35 different health posts in the region had received eye care training, with 72.9% of PHCWs unable to diagnose MK. At the same time as improving access to primary care, there needs to be significant investment in training primary healthcare workers in ophthalmology to prevent missed diagnoses of ophthalmic emergencies such as MK, and to start appropriate treatment promptly (and/or refer as appropriate); a third opportunity for intervention. These measures should further help reduce the delay for MK patients accessing appropriate care. Our results, in agreement with previous studies, suggest that patients tend to seek help promptly after developing symptoms, likely due to the pain and poor vision (9). The immediate actions of who sees them first have a significant bearing on any delay to appropriate facilities and their overall prognosis. If patients attended a facility with appropriate MK diagnostic facilities (microscopy and culture as a minimum), experienced clinicians and available treatment (quinolone antibiotics and antifungals such as natamycin) within 1 week of symptom onset, the outcome is likely to be much better (11, 12, 15).

SCEH operates on a hub-and-spoke model, similar to other tertiary ophthalmic centers, with ECCs acting as a primary-care level facility with trained ophthalmic clinicians who can see and treat a set number of conditions, referring more complex cases to the SCEH hub, including ophthalmic emergencies such as MK. ECCs provide a very valuable service by improving access to ophthalmic care for patients. Although ECCs were the only primary-level provider to refer patients directly to SCEH, only 26/150 (17.3%) of ECC attendances were referred to SCEH directly, with the remainder self-referring later. Given the time-critical nature of MK and the need for additional diagnostic facilities not available at ECCs, these patients should have all been referred directly. Direct referral reduces any delay and can easily be arranged by hospital transport if necessary. Strengthening this referral pathway is a fourth opportunity for intervention. In addition, ECCs need to be fully integrated into the primary care system in the region, and expanded to areas where they are lacking, to help improve access further.

### Strengths and limitations

We believe this is the first study from Asia to systematically investigate the care-seeking journey of patients with MK and examine factors that influence this. It has a large sample from a wide geographic area, which affords examination of these questions. It highlights that more severe disease is associated with delayed and complex presentation journeys, and the harms linked to these. It points to current gaps in the health system in Nepal and northern India, and how these can be addressed to potentially improve outcomes. Furthermore, it includes high quality microbiology in addition to *in vivo* confocal microscopy to identify the causative organism.

There are several limitations. First, there may be incomplete recall by patients of medications used and costs incurred; not every patient had medications or receipts with them at the time of presentation. Second, we did not conduct any formal qualitative research as part of this study, which may have provided insights into the journey and choice of facilities used. Although informal conversations with participants did highlight some possible reasons for delay these were not collected in a systematic manner and therefore not included in this analysis. We are currently conducting qualitative research into the knowledge and beliefs of pharmacists and traditional healers to explore their dispensing practices to identify approaches to influence their practice to improve patient care. Third, we did not analyse how final clinical outcomes may be related to delay as this study formed part of recruitment for a clinical trial that only included patients with fungal keratitis and randomized patients to two different treatments. Fourthly, there may have been some additional delays due to extrinsic factors such as COVID-19 and local flooding during the monsoon. However, there was no significant difference between the interval from symptom onset to presentation for patients attending before or after the start of COVID-19 restrictions in March 2020. Given that this was an observational study, causality of any relationships was precluded, so we are unable to establish in this study if delayed presentation led to worse clinical outcomes, although this has been previously reported in other settings (9, 10, 15). Finally, there is a degree of selection bias within this study as all sampling occurs at the tertiary level hospital, meaning that any cases of MK that were managed in the community and subsequently improved were not captured in this study. For a more accurate assessment, all health facilities that managed MK would need to be studied, but this would not be feasible given the large geographical area across two countries with many primary and secondary health facilities including pharmacies and traditional healers.

## Conclusion

We found that most patients attending a tertiary eye hospital with microbial keratitis did not present directly. They often visited multiple health facilities, requiring many journeys, leading to increased costs, delays, and more advanced disease at presentation. This highlights a number of factors that are worthy of further, more detailed investigation, that if improved, could reduce delay and improve outcomes: appropriate early treatment with antibiotics and avoiding harmful treatment (e.g., steroids); appropriate early, direct referral to an eye hospital with appropriate diagnostic facilities and treatments whilst strengthening the referral to the main hospital from satellite clinics; improving access to eye care professionals; and, educating patients to attend an eye specialist directly. Reducing delay, combined with improved diagnostics and more effective treatment, will help improve outcomes for the millions of patients who develop microbial keratitis annually.

## Data availability statement

The raw data supporting the conclusions of this article will be made available by the authors, without undue reservation.

## Ethics statement

The studies involving human participants were reviewed and approved by London School of Hygiene & Tropical Medicine Ethics Committee (Ref. 14841) and Nepal Health Research Council Ethical Review Board (Ref. 1937). The patients/participants provided their written informed consent to participate in this study.

## Author contributions

JH and MB: conceptualization. JH, SA, DM, and MB: methodology. JH and DM: formal analysis. JH, RY, SD, PC, and AL: investigation. AR and SS: resources. JH, SD, PC, and AL: data curation. JH: writing—original draft preparation. VH, AL, and MB: supervision. JH, SD, and AR: project administration. MB: funding acquisition. All co-authors writing: review and editing. All authors have read and agreed to the published version of the manuscript.

## Funding

This research was funded through a Senior Research Fellowship to MB from the Wellcome Trust (207472/Z/17/Z). The funders had no role in study design, data collection and analysis, decision to publish, or preparation of the manuscript.

## Collaborators

Sagarmatha Choudhary Eye Hospital: Abhishek Roshan (Hospital Manager); Sanjay Kumar Singh (Medical Superintendent); Reena Yadav (Primary Investigator); Sandip Das Sanyam (Study Co-Ordinator); Pankaj Chaudhary (Microbiologist); Rabi Shankar Sah, Kamlesh Yadav (Investigators); Ram Narayan Bhandari, Aasha Chaudhary, Sharban Man-dal (Eye Health Workers); Raja Ram Mahato (Randomization Administrator and Logistics); Lalita Rajbanshi (Laboratory Assistant); Ramesh Sah, Arvind Ray, Sachindra Kamti (Optome-trists); Avinash Chaudhary (Ophthalmic Assistant); Padma Narayan Chaudhary (Hospital Chairman); Suresh Singh, Ravi Pant, Rakesh Singh (Hospital Management); Ram Kumar Jha (Ophthalmic Assistant, Rajbiraj ECC). Nepal Netra Jyoti Sangh: Sailesh Kumar Mishra (Executive Director); Sabita KC (Board Secretary); Ranjan Shah (Programme Associate); Jaganath Dhital (Assistant). Eastern Region Eye Care Programme: Sanjay Kumar Singh (Programme Director). Janakpur Eye Hospital: Hemchandra Jha (Medical Superintendent); Mahesh Yadav (Investiga-tor); Rudal Prasad Sah (Ophthalmic Assistant). London School of Hygiene & Tropical Medicine: Jeremy Hoffman (Primary Investigator); Matthew Burton (Chief Investigator); Astrid Leck (Mi-crobiologist); David Macleod, Helen Weiss (Statisticians); Victor Hu (Investigator); Sarah O'Regan (Administrator).

## Conflict of interest

The authors declare that the research was conducted in the absence of any commercial or financial relationships that could be construed as a potential conflict of interest.

## Publisher's note

All claims expressed in this article are solely those of the authors and do not necessarily represent those of their affiliated organizations, or those of the publisher, the editors and the reviewers. Any product that may be evaluated in this article, or claim that may be made by its manufacturer, is not guaranteed or endorsed by the publisher.

## References

[1] Whitcher JP Srinivasan M Upadhyay MP. Corneal blindness: a global perspective. Bull World Health Organiz. (2001) 79:214–21. PMC2566379 11285665

[2] Ung L Bispo PJM Shanbhag SS Gilmore MS Chodosh J. The persistent dilemma of microbial keratitis: global burden, diagnosis, and antimicrobial resistance. Surv Ophthalmol. (2019) 64:255–71. 10.1016/j.survophthal.2018.12.003 30590103 PMC7021355

[3] Arunga S Wiafe G Habtamu E Onyango J Gichuhi S Leck A. The impact of microbial keratitis on quality of life in Uganda. BMJ Open Ophthalmol. (2019) 4:e000351. 10.1136/bmjophth-2019-000351 31909191 PMC6936408

[4] Hossain P. Microbial keratitis-the true costs of a silent pandemic? Eye. (2021) 35:2071–2. 10.1038/s41433-020-01360-6 33594242 PMC8302703

[5] Ung L Acharya NR Agarwal T Alfonso EC Bagga B Bispo PJ. Infectious corneal ulceration: a proposal for neglected tropical disease status. Bull World Health Organ. (2019) 97:854–6. 10.2471/BLT.19.232660 31819296 PMC6883276

[6] Upadhyay MP Karmacharya PC Koirala S Shah DN Shakya S Shrestha JK. The bhaktapur eye study: ocular trauma and antibiotic prophylaxis for the prevention of corneal ulceration in nepal. Brit J Ophthalmol. (2001) 85:388–92. 10.1136/bjo.85.4.388 11264124 PMC1723912

[7] Hoffman JJ Burton MJ Leck A. Mycotic keratitis—a global threat from the filamentous fungi. J Fungi. (2021) 7:273. 10.3390/jof7040273 33916767 PMC8066744

[8] Sitoula RP Singh S Mahaseth V Sharma A Labh R. Epidemiology and etiological diagnosis of infective keratitis in eastern region of Nepal. Nepal J Ophthalmol. (2015) 7:10–5. 10.3126/nepjoph.v7i1.13146 26695600

[9] Arunga S Kintoki GM Gichuhi S Onyango J Newton R Leck A. Delay along the care seeking journey of patients with microbial keratitis in Uganda. Ophthalmic Epidemiol. (2019) 26:311–20. 10.1080/09286586.2019.1616775 31088316 PMC7446038

[10] Arunga S Kintoki GM Mwesigye J Ayebazibwe B Onyango J Bazira J. Epidemiology of microbial keratitis in uganda: a cohort study. Ophthalmic Epidemiol. (2020) 27:121–31. 10.1080/09286586.2019.1700533 31830848 PMC7446037

[11] Prajna NV Krishnan T Mascarenhas J Srinivasan M Oldenburg CE Toutain-Kidd CM. Predictors of outcome in fungal keratitis. Eye. (2012) 26:1226–31. 10.1038/eye.2012.99 22744392 PMC3443844

[12] Getshen K Srinivasan M Upadhyay MP Priyadarsini B Mahalaksmi R Whitcher JP. Corneal ulceration in south east Asia. I: a model for the prevention of bacterial ulcers at the village level in rural bhutan. Brit J Ophthalmol. (2006) 90:276–8. 10.1136/bjo.2005.076083 16488943 PMC1856957

[13] Maung N Thant CC Srinivasan M Upadhyay MP Priyadarsini B Mahalakshmi R. Corneal ulceration in south east Asia. Ii: a strategy for the prevention of fungal keratitis at the village level in burma. Brit J Ophthalmol. (2006) 90:968–70. 10.1136/bjo.2006.094706 16707522 PMC1857195

[14] Bajracharya L Bade AR Gurung R Dhakhwa K. Demography, risk factors, and clinical and microbiological features of microbial keratitis at a tertiary eye hospital in Nepal. Clin Ophthalmol. (2020) 14:3219–26. 10.2147/OPTH.S266218 33116372 PMC7567540

[15] Burton MJ Pithuwa J Okello E Afwamba I Onyango JJ Oates F. Microbial keratitis in east africa: why are the outcomes so poor? Ophthalmic Epidemiol. (2011) 18:158–63. 10.3109/09286586.2011.595041 21780874 PMC3670402

[16] Hoffman JJ Yadav R Das Sanyam S Chaudhary P Roshan A Singh SK. Topical chlorhexidine 0.2% versus topical natamycin 5% for fungal keratitis in Nepal: rationale and design of a randomised controlled non-inferiority trial. BMJ Open. (2020) 10:e038066. 10.1136/bmjopen-2020-038066 32998924 PMC7528427

[17] Srinivasan M Mascarenhas J Rajaraman R Ravindran M Lalitha P Glidden DV. Corticosteroids for bacterial keratitis: the steroids for corneal ulcers trial (Scut). Arch Ophthalmol. (2012) 130:143–50. 10.1001/archophthalmol.2011.315 21987582 PMC3830549

[18] Group A-REDSR. The age-related eye disease study (areds): design implications. Areds Report No. 1. Control Clin Trials. (1999) 20:573–600. 10.1016/S0197-2456(99)00031-8 10588299 PMC1473211

[19] Prajna NV Krishnan T Mascarenhas J Rajaraman R Prajna L Srinivasan M. The mycotic ulcer treatment trial: a randomized trial comparing natamycin vs voriconazole. JAMA Ophthalmol. (2013) 131:422–9. 10.1001/jamaophthalmol.2013.1497 23710492 PMC3769211

[20] Chidambaram JD Prajna NV Larke N Macleod D Srikanthi P Lanjewar S. *In vivo* confocal microscopy appearance of fusarium and aspergillus species in fungal keratitis. Brit J Ophthalmol. (2017) 101:1119–23. 10.1136/bjophthalmol-2016-309656 28043985 PMC5537506

[21] Chidambaram JD Prajna NV Larke NL Palepu S Lanjewar S Shah M. Prospective study of the diagnostic accuracy of the in vivo laser scanning confocal microscope for severe microbial keratitis. Ophthalmology. (2016) 123:2285–93. 10.1016/j.ophtha.2016.07.009 27538797 PMC5081072

[22] Google Inc,. Google My Maps (2021). Available online at: https://www.google.com/maps/about/mymaps/ (accessed September 6, 2021).

[23] Bert BK Rekha H Percy MK. Ocular injuries and eye care seeking patterns following injuries among cocoa farmers in ghana. Afr Health Sci. (2016) 16:255–65. 10.4314/ahs.v16i1.34 27358640 PMC4915394

[24] Lam Y Broaddus ET Surkan PJ. Literacy and healthcare-seeking among women with low educational attainment: analysis of cross-sectional data from the 2011 nepal demographic and health survey. Int J Equity Health. (2013) 12:95. 10.1186/1475-9276-12-95 24330671 PMC3878725

[25] Puri LR Shrestha G. Microbial keratitis: a five years retrospective clinical study in tertiary eye hospital of eastern region of nepal. J Kathmandu Med Coll. (2017) 4:118–25. 10.3126/jkmc.v4i4.18252

[26] Al-Attas AH Williams CD Pitchforth EL O'Callaghan CO Lewallen S. Understanding delay in accessing specialist emergency eye care in a developing country: eye trauma in tanzania. Ophthalmic Epidemiol. (2010) 17:103–12. 10.3109/09286580903453522 20132093

[27] Gichuhi S Kabiru J M'Bongo Zindamoyen A Rono H Ollando E Wachira J. Delay along the care-seeking journey of patients with ocular surface squamous neoplasia in kenya. BMC Health Serv Res. (2017) 17:485. 10.1186/s12913-017-2428-4 28705204 PMC5512725

[28] Bronsard A Geneau R Shirima S Courtright P Mwende J. Why are children brought late for cataract surgery? Qualitative findings from tanzania. Ophthalmic Epidemiol. (2008) 15:383–8. 10.1080/09286580802488624 19065431

[29] Shah H Radhakrishnan N Ramsewak S Chiu S Joseph S Rose-Nussbaumer J. Demographic and socioeconomic barriers and treatment seeking behaviors of patients with infectious keratitis requiring therapeutic penetrating keratoplasty. Ind J Ophthalmol. (2019) 67:1593–8. 10.4103/ijo.IJO_1821_18 31546487 PMC6786147

[30] Fletcher AE Donoghue M Devavaram J Thulasiraj RD Scott S Abdalla M. Low uptake of eye services in rural India: a challenge for programs of blindness prevention. Arch Ophthalmol. (1999) 117:1393–9. 10.1001/archopht.117.10.1393 10532449

[31] Moussa G Hodson J Gooch N Virdee J Penaloza C Kigozi J. Calculating the economic burden of presumed microbial keratitis admissions at a tertiary referral centre in the Uk. Eye. (2021) 35:2146–54. 10.1038/s41433-020-01333-9 33288899 PMC8302743

[32] Keay L Edwards K Dart J Stapleton F. Grading contact lens-related microbial keratitis: relevance to disease burden. Optom Vis Sci. (2008) 85:531–7. 10.1097/OPX.0b013e31817dba2e 18594345

[33] Burn H Puri L Roshan A Singh SK Burton MJ. Primary eye care in eastern nepal. Ophthalmic Epidemiol. (2019) 22:1–12. 10.1080/09286586.2019.1702217 31842661 PMC7114913

