# Supplementary material for: Delay in accessing definitive care for patients with microbial keratitis in Nepal
Source: Front Med (Lausanne). 2022 Jul 22;9:915293. doi: 10.3389/fmed.2022.915293 (PMC9354956; doi:10.3389/fmed.2022.915293)
Supplement: Supplementary file 1 [file Data_Sheet_1.PDF]

Supplementary Material 1: Risk factors investigated for an association with delayed presentation or indirect attendance

**Age**	**Previous treatment[1]**
**Sex (being female)**	**Previous steroids[1]**
**Marital status (being married)**	**Previous antibiotics[1]**
**Farmer Occupation**	**Previous antifungals[1]**
**Distance to SCEH (km)**	**Previous other topical medication[1]**
**Distance from nearest health centre (km)**	**Previous systemic medication[1]**
**Positive history of trauma**	**Used TEM**
**Education Status**	**Number of journeys[1]**
**Country of residence (India)**	**Visiting one or more facilities prior to SCEH[1]**

[1]Variables only investigated for delayed presentation. All other variables were investigated for associations with delayed presentation or indirect attendance.

SCEH, Sagarmatha Choudhary Eye Hospital; TEM, traditional eye medicine

Supplementary Table 1: Rates of trauma between farmers and non-farmers

	**Non-farmer**		**Farmer**		**Total (N=643)**	
	n	(%)	n	(%)	n	(%)
**History of trauma**	190	(61.1)	136	(40.1)	326	(50.7)
**No history of trauma**	121	(38.9)	196	(59.0)	317	(49.3)

P <0.001 (Fisher's exact)

Supplementary Table 2: Multivariable logistic regression analysis of factors associated with direct presentation to the eye hospital for patients resident in Nepal and patients resident in India.

	**Patients resident in Nepal (n=371)**			**Patients resident in India (n=271)**		
	aOR	(95% CI)	P value	aOR	(95% CI)	P value
**Age**	1.004	(0.987-1.022)	0.625	1.009	(0.983-1.035)	0.499
**Sex (being female)**	1.262	(0.771-2.066)	0.355	0.872	(0.436-1.756)	0.700
**Farmer Occupation**	1.372	(0.851-2.212)	0.194	1.521	(0.758-3.054)	0.238
**Distance to SCEH (km)**						
0-5	-	-	-	-	-	-
>5-20	0.611	(0.223-1.654)	0.332	N/A		
>20-50	0.175	(0.065-0.470)	0.001	1.295	(0.490-3.421)	0.602
>50-100	0.243	(0.069-0.855)	0.028	1.133	(0.498-2.576)	0.766
>100	0.197	(0.041-0.953)	0.043	1		

Supplementary Table 3: Multivariable ordinal logistic regression analysis of factors associated with delayed presentation to the eye hospital for patients resident in Nepal and patients resident in India

	**Patients resident in Nepal (n=371)**			**Patients resident in India (n=271)**		
	aOR	(95% CI)	P value	aOR	(95% CI)	P value
**Age**	1.009	(0.993-1.025)	0.243	1.000	(0.985-1.017)	0.935
**Sex (being female)**	1.186	(0.756-1.862)	0.458	1.023	(0.656-1.596)	0.921
**Farmer Occupation**	1.068	(0.693-1.645)	0.765	1.237	(0.791-1.938)	0.352
**Distance to SCEH (km)**						
0-5						
>5-20	0.844	(0.279-2.552)	0.764	0.585	(0.029-11.91)	0.727
>20-50	1.948	(0.681-5.571)	0.214	N/A		
>50-100	3.860	(1.109-13.44)	0.034	1.606	(0.081-31.83)	0.756
>100	7.050	(1.626-30.56)	0.009	2.648	(0.131-53.68)	0.526
